# Author Correction: Synergistic H&E and IHC image analysis by AI predicts cancer biomarkers and survival outcomes in colorectal and breast cancer

**DOI:** 10.1038/s43856-025-01097-x

**Published:** 2025-10-24

**Authors:** Yating Cheng, Norsang Lama, Ming Chen, Eghbal Amidi, Mohammadreza Ramzanpour, Md Ashequr Rahman, Joanne Xiu, Anthony Helmstetter, Lauren Dickman, Jennifer R. Ribeiro, Hassan Ghani, Matthew Oberley, David Spetzler, George W. Sledge

**Affiliations:** Caris Life Sciences, Phoenix, AZ USA

**Keywords:** Tumour immunology, Computational biology and bioinformatics, Tumour biomarkers

Correction to: *Communications Medicine* 10.1038/s43856-025-01045-9, published online 1 August 2025

In this article the author name Eghbal Amidi was incorrectly listed as Egbhal Amidi. The original article has been corrected.

