# Supplementary material for: Synergistic H&E and IHC image analysis by AI predicts cancer biomarkers and survival outcomes in colorectal and breast cancer
Source: Commun Med (Lond). 2025 Aug 1;5:328. doi: 10.1038/s43856-025-01045-9 (PMC12317095; doi:10.1038/s43856-025-01045-9)

Supplementary Information

Synergistic H&E and IHC Image Analysis by AI Predicts Cancer Biomarkers and Survival Outcomes in Colorectal and Breast Cancer

Yating Cheng[1]*, Norsang Lama[1]*, Ming Chen[1], Egbhal Amidi[1], Mohammadreza Ramzanpour[1], Md Ashequr Rahman[1], Joanne Xiu[1], Anthony Helmstetter[1], Lauren Dickman[1], Jennifer R. Ribeiro[1], Hassan Ghani[1], Matthew Oberley[1], David Spetzler[1], George W. Sledge[1]

[1]Caris Life Sciences, Phoenix, Arizona.

* These authors contributed equally to this work

Corresponding Authors

George W. Sledge, Jr., MD and Ming Chen, PhD

Caris Life Sciences

4550 S. 44th Pl.

Phoenix, AZ 85040

Phone: (602) 464-7500

Emails: gsledge@carisls.com; mchen@carisls.com

Supplementary Table 1. Biomarker status distribution for modeling dataset

	CRC (MMRd), N (%)	CRC (MSI), N (%)	BRCA (PD-L1), N (%)
**Biomarker Status**	Deficient: 1262 (6%)	MSI: 1375 (7%)	Positive: 2366 (16%)
	Proficient: 19091(94%)	MSS: 19035 (93%)	Negative: 12341 (84%)

Supplementary Table 2. Biomarker status distribution for holdout dataset

	CRC (MMRd), N (%)	CRC (MSI), N (%)	BRCA (PD-L1), N (%)
**Biomarker Status**	Deficient: 280(60%)	MSI: 287 (61%)	Positive: 265 (57%)
	Proficient: 187(40%)	MSS: 182 (39%)	Negative: 201 (43%)

Supplementary Table 3. Scanner type distribution for modeling dataset

	CRC (MMRd)			CRC (MSI)			BRCA (PD-L1)		
	**H&E**	**IHC**	**Difference (%)[a]**	**H&E**	**IHC**	**Difference (%)[a]**	**H&E**	**IHC**	**Difference (%)[a]**
**Phillips**	14041	16892	25.67	13888	17134	26.9	9035	4825	38.61
**Leica**	6312	3461		6522	3276		5672	9882	

[a]% of cases in which H&E and IHC images were scanned by different scanners.

Supplementary Table 4. Scanner type distribution for holdout dataset

	CRC (MMRd)			CRC (MSI)			BRCA (PD-L1)		
	**H&E**	**IHC**	**Difference (%)[a]**	**H&E**	**IHC**	**Difference (%)[a]**	**H&E**	**IHC**	**Difference (%)[a]**
**Phillips**	426	432	11.13	428	434	11.09	331	300	29.83
**Leica**	41	35		41	35		135	166	

[a]% of cases in which H&E and IHC images were scanned by different scanners.

Supplementary Table 5. Specimen site distribution for modeling dataset

	CRC (MMRd)			CRC (MSI)			BRCA (PD-L1)		
	H&E	IHC	Difference (%)[a]	H&E	IHC	Difference (%)[a]	H&E	IHC	Difference (%)[a]
Primary	12039	12076	0.7	12519	12548	0.66	5800	5792	0.78
Metastatic	8142	8112		7709	7684		8679	8688	
Unknown	172	165		182	178		228	227	

[a]% of cases in which H&E and IHC images were collected from different specimen sites.

Supplementary Table 6. Specimen site distribution for holdout dataset

	CRC (MMRd)			CRC (MSI)			BRCA (PD-L1)		
	H&E	IHC	Difference (%)[a]	H&E	IHC	Difference (%)[a]	H&E	IHC	Difference (%)[a]
Primary	286	286	0	287	287	0	268	270	0.86
Metastatic	173	173		174	174		188	187	
Unknown	8	8		8	8		10	9	

[a]% of cases in which H&E and IHC images were collected from different specimen sites.

Supplementary Table 7. Summary of the statistical analyses performed on the clinical outcomes with respect to time-on-treatment (TOT)

		**CRC (MMRd)[b]**	**CRC (MSI)[c]**	**BRCA (PD-L1)[d]**
**Pathologist IHC/NGS call**	Neg/Pos Median (Months)	2.79/8.97	2.76/9.3	5.06/5.98
	HR [95% CI]	0.463 [0.365-0.587]	0.437 [0.344-0.555]	0.785 [0.629-0.979]
	p-value[a]	<0.001	<0.001	0.032
**H&E**	Neg/Pos Median (Months)	3.45/7.82	3.45/7.82	4.01/6.18
	HR [95% CI]	0.552 [0.436-0.698]	0.577 [0.456-0.731]	0.671 [0.525-0.858]
	p-value[a]	<0.001	<0.001	0.001
**IHC**	Neg/Pos Median (Months)	3.45/8.25	3.45/8.28	4.17/6.15
	HR [95% CI]	0.577 [0.456-0.73]	0.636 [0.502-0.805]	0.784 [0.62-0.993]
	p-value[a]	<0.001	<0.001	0.044
**H&E + IHC**	Neg/Pos Median (Months)	3.42/8.97	3.29/8.97	4.14/6.15
	HR [95% CI]	0.506 [0.4-0.641]	0.535 [0.421-0.679]	0.741 [0.583-0.942]
	p-value[a]	<0.001	<0.001	0.014

[a]p-values from the log-rank test comparing biomarker status (positive vs. negative) as determined by the pathologist's IHC/NGS call and as predicted by each model.

[b]For MMRd biomarker, 'neg' indicates proficient MMR (MMRp) and 'pos' indicates deficient MMR (MMRd).

[c]For the MSI biomarker, 'neg' represents MSS and 'pos' represents MSI-H.

[d]For the PD-L1 biomarker, 'neg' signifies CPS < 10 and 'pos' signifies CPS ^{3}10.

Abbreviations: BRCA = breast cancer, CI = confidence interval, CRC = colorectal cancer, H&E = hematoxylin & eosin, HR = hazard ratio, IHC = immunohistochemistry, MMRd = mismatch repair deficiency, MSI = microsatellite instability, NGS = next-generation sequencing

Supplementary Table 8. Summary of the statistical analyses performed on the clinical outcomes with respect to overall survival (OS)

		CRC (MMRd)[b]	CRC (MSI)[c]	BRCA (PD-L1)[d]
**Pathologist IHC/NGS call**	Neg/Pos Median (Months)	17.03/Inf	15.91/Inf	Inf/Inf
	HR [95% CI]	0.398 [0.282-0.562]	0.366 [0.258-0.52]	0.882 [0.626-1.241]
	p-value[a]	<0.001	<0.001	0.47
**H&E**	Neg/Pos Median (Months)	19.1/Inf	19.79/Inf	15.68/Inf
	HR [95% CI]	0.41 [0.289-0.581]	0.447 [0.315-0.634]	0.511 [0.358-0.729]
	p-value[a]	<0.001	<0.001	<0.001
**IHC**	Neg/Pos Median (Months)	19.1/Inf	19.79/Inf	16.21/Inf
	HR [95% CI]	0.386 [0.269-0.554]	0.437 [0.307-0.624]	0.625 [0.44-0.889]
	p-value[a]	<0.001	<0.001	0.009
**H&E + IHC**	Neg/Pos Median (Months)	17.03/Inf	14.63/Inf	16.21/Inf
	HR [95% CI]	0.38 [0.267-0.539]	0.348 [0.245-0.494]	0.601 [0.421-0.858]
	p-value[a]	<0.001	<0.001	0.005

[a]p-values from the log-rank test comparing biomarker status (positive vs. negative) as determined by the pathologist's IHC/NGS call and as predicted by each model.

[b]For MMRd biomarker, 'neg' indicates proficient MMR (MMRp) and 'pos' indicates deficient MMR (MMRd).

[c]For the MSI biomarker, 'neg' represents MSS and 'pos' represents MSI-H.

[d]For the PD-L1 biomarker, 'neg' signifies CPS < 10 and 'pos' signifies CPS ^{3}10.

Abbreviations: BRCA = breast cancer, CI = confidence interval, CRC = colorectal cancer, H&E = hematoxylin & eosin, HR = hazard ratio, IHC = immunohistochemistry, MMRd = mismatch repair deficiency, MSI = microsatellite instability, NGS = next-generation sequencing

Supplementary Table 9. Comparison of AUROC across models utilizing different feature encoders

	CRC-MMR			CRC-MSI			BRCA-PD-L1		
	**HE**	**IHC**	**Duet**	**HE**	**IHC**	**Duet**	**HE**	**IHC**	**Duet**
**CTransPath**	0.922 ±0.009	0.947 ±0.008	0.967 ±0.006	0.939 ±0.007	0.952 ±0.006	0.973 ±0.005	0.866 ±0.008	0.959 ±0.004	0.957 ±0.004
**Virchow**	0.927 ±0.014	0.948 ±0.010	0.960 ±0.006	0.943 ±0.010	0.950 ±0.005	0.970 ±0.008	0.871 ±0.005	0.959 ±0.005	0.958 ±0.004
**Virchow2**	0.946 ±0.011	0.965 ±0.006	0.974 ±0.007	0.961 ±0.009	0.968 ±0.009	0.985 ±0.005	0.886 ±0.011	0.963 ±0.006	0.962 ±0.004
**UNI**	0.940 ±0.010	0.961 ±0.012	0.973 ±0.007	0.956 ±0.010	0.965 ±0.012	0.981 ±0.008	0.880 ±0.009	0.963 ±0.005	0.961 ±0.005
**H-Optimus-0**	0.945 ±0.013	0.958 ±0.010	0.973 ±0.007	0.961 ±0.009	0.960 ±0.016	0.981 ±0.007	0.883 ±0.008	0.960 ±0.005	0.958 ±0.007

Supplementary Fig. 1. Distribution of prediction probabilities. (a) Displays the prediction probability distribution for mismatch repair deficient (MMRd) using three different models: hematoxylin & eosin (H&E) only, immunohistochemistry (IHC) only, and a combined H&E + IHC model. **(b)** Shows the prediction probability distribution for microsatellite instability (MSI) across the same three models as in **(a)**.

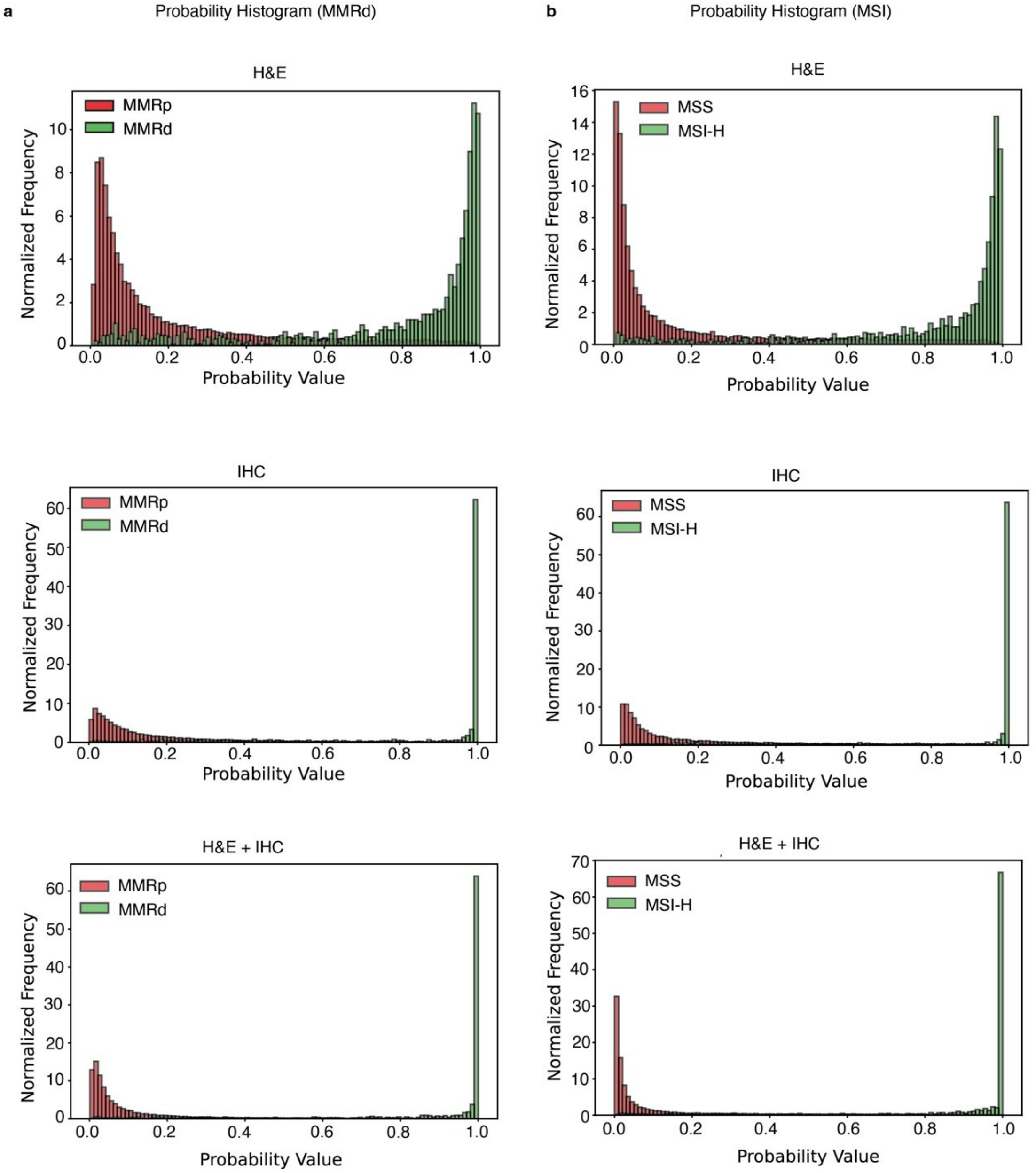

Supplementary Fig. 2. **Visualization of attention and classification scores for colorectal cancer (CRC) specimens.** Hematoxylin & eosin (H&E) and immunohistochemistry (IHC) images are shown for mismatch repair proficient (MMRp) **(a)**, microsatellite instability-high (MSI-H) **(b)** and microsatellite stable (MSS) specimens **(c)**.

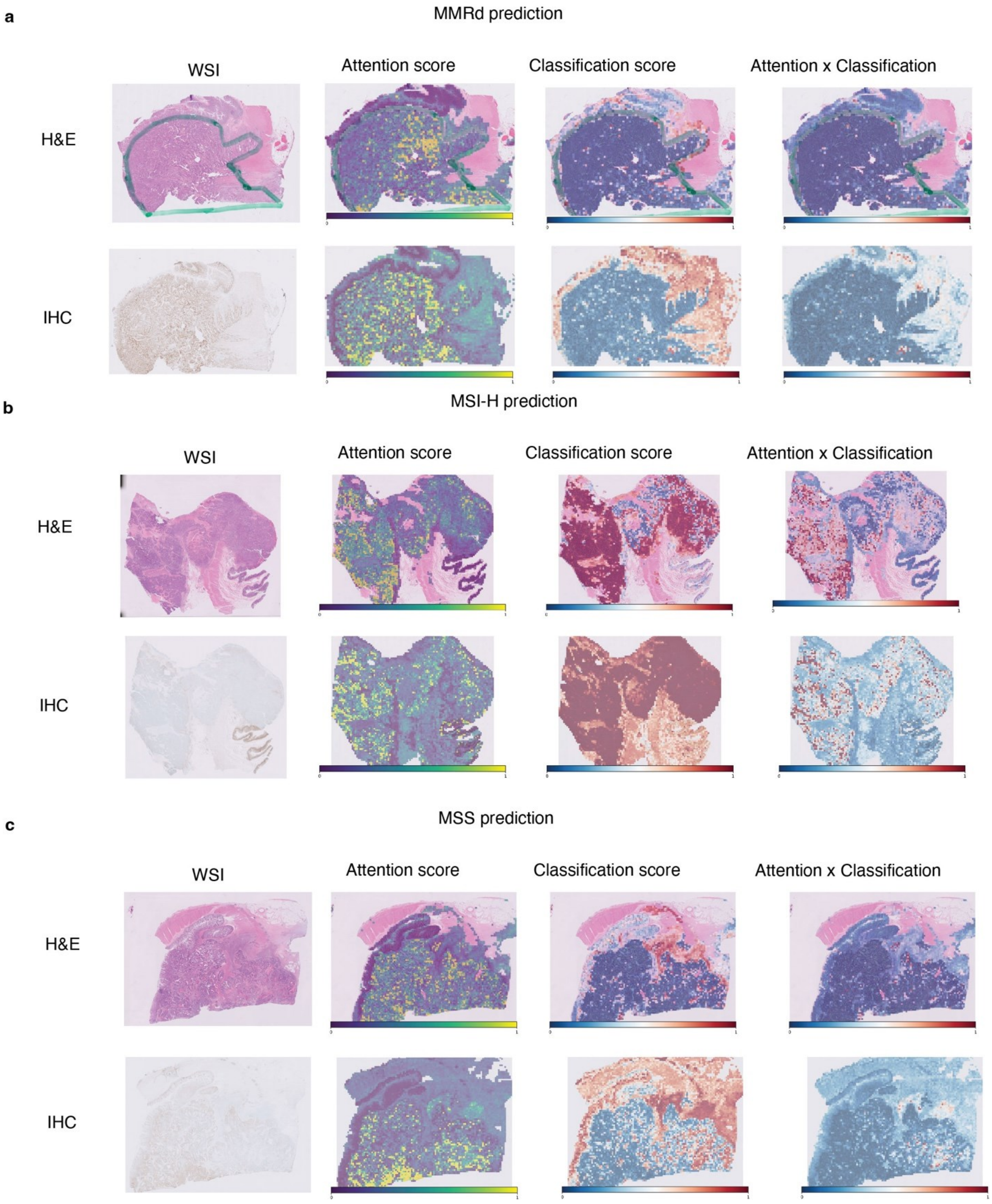

Supplementary Fig. 3. Visualization of attention and classification scores for colorectal cancer (CRC) specimens that were misclassified with respect to MMRd status. Hematoxylin & eosin (H&E) and immunohistochemistry (IHC) images are shown for an MMRd case predicted to be MMRp (**a**) and an MMRp case predicted to be MMRd (**b**).

a MMRd predicted to be MMRp

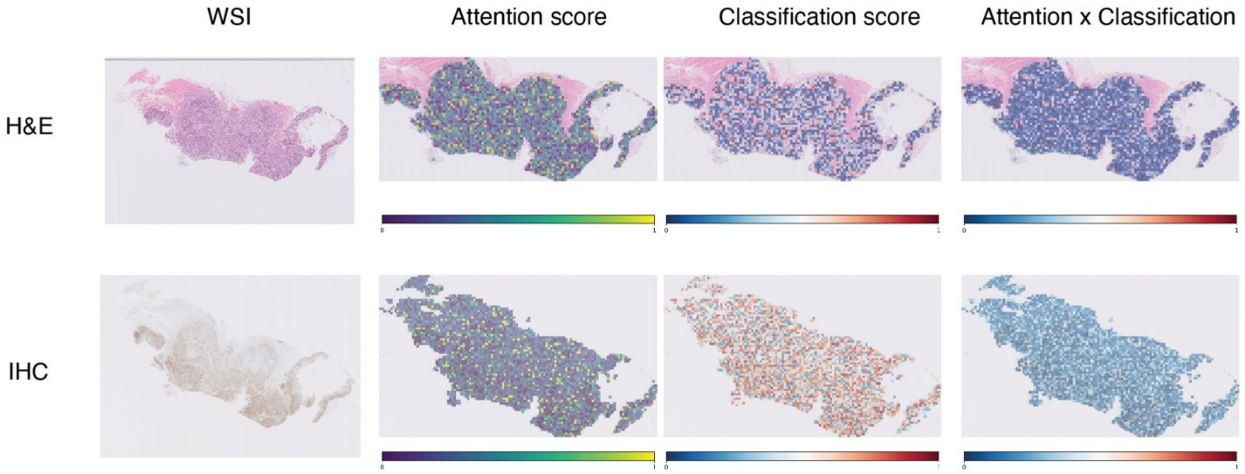

b MMRp predicted to be MMRd

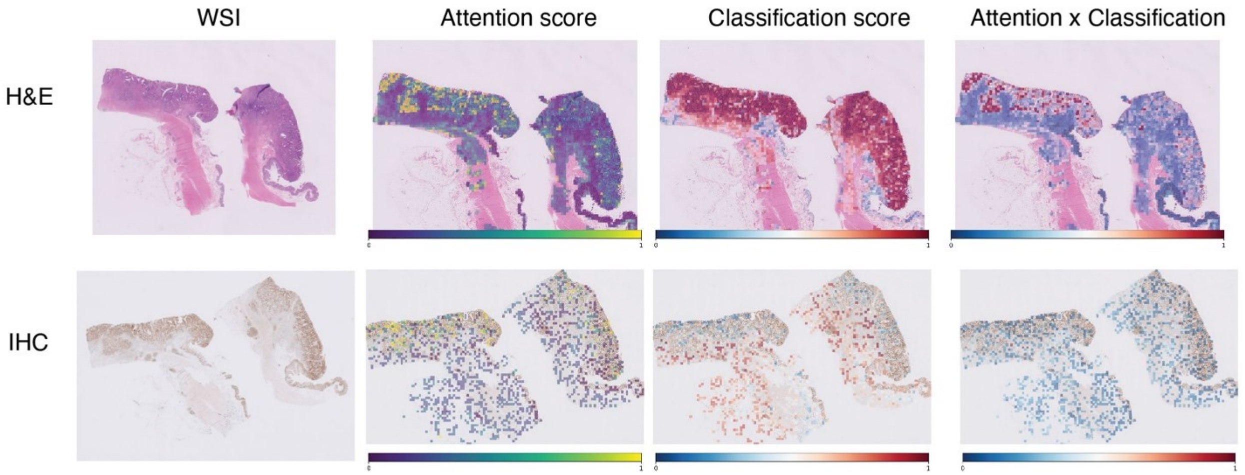

Supplementary Fig. 4. Visualization of attention and classification scores for colorectal cancer (CRC) specimens that were misclassified with respect to MSI status. Hematoxylin & eosin (H&E) and immunohistochemistry (IHC) images are shown for an MSI-H case predicted to be MSS (**a**) and an MSS case predicted to be MSI-H (**b**).

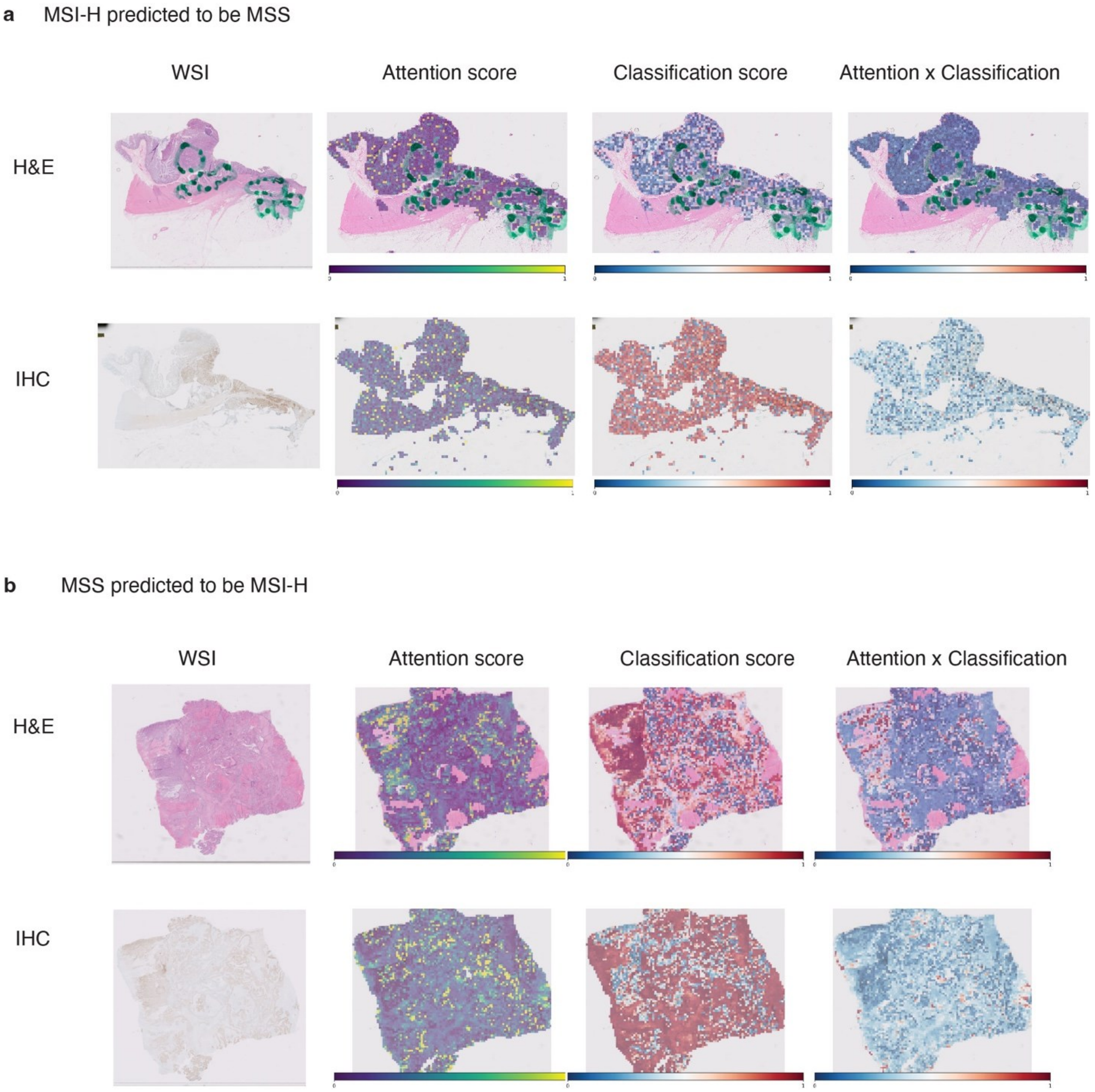

Supplementary Fig. 5 Visualization of attention and classification scores for breast cancer (BRCA) specimens. Hematoxylin & eosin (H&E) and immunohistochemistry (IHC) images are shown for PD-L1-positive specimens.

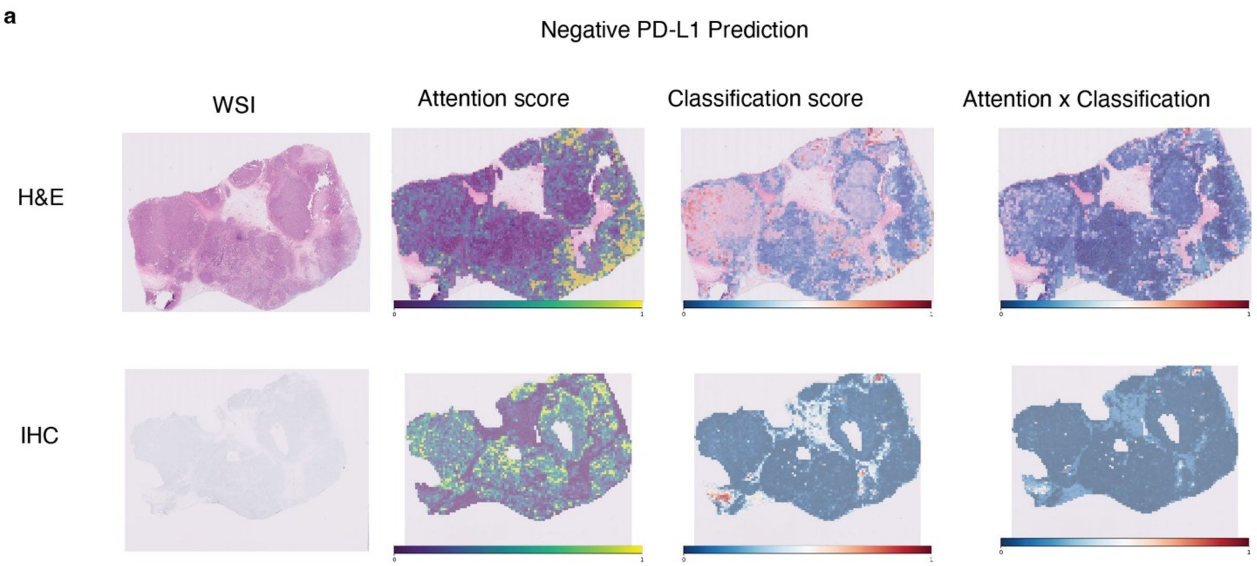

Supplementary Fig. 6 Visualization of attention and classification scores for breast cancer (BRCA) specimens that were misclassified with respect to PD-L1 status. Hematoxylin & eosin (H&E) and immunohistochemistry (IHC) images are shown for a PD-L1-negative case predicted to be positive (**a**) and a PD-L1-positive case predicted to be negative (**b**).

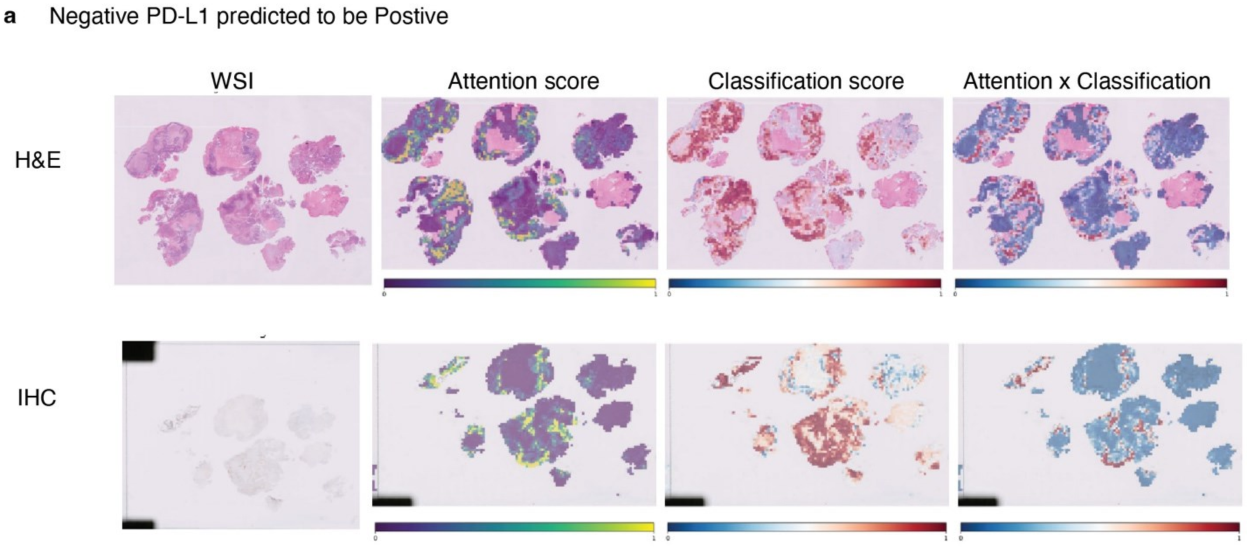

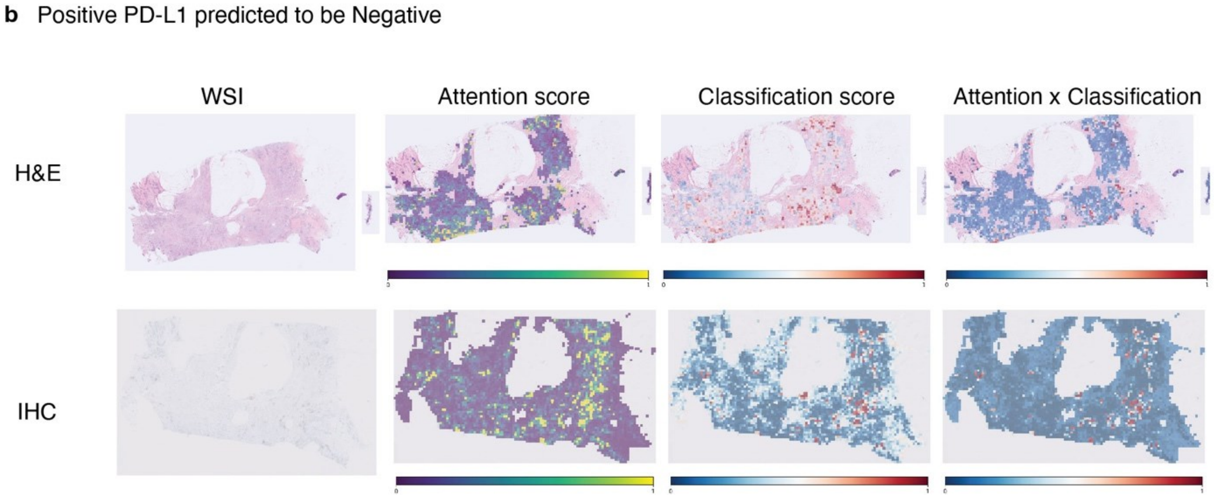

Supplementary Fig. 7 Distribution of prediction probabilities. Displays the prediction probability distribution for PD-L1 using three different models: hematoxylin & eosin (H&E) only, immunohistochemistry (IHC) only, and a combined H&E + IHC model.

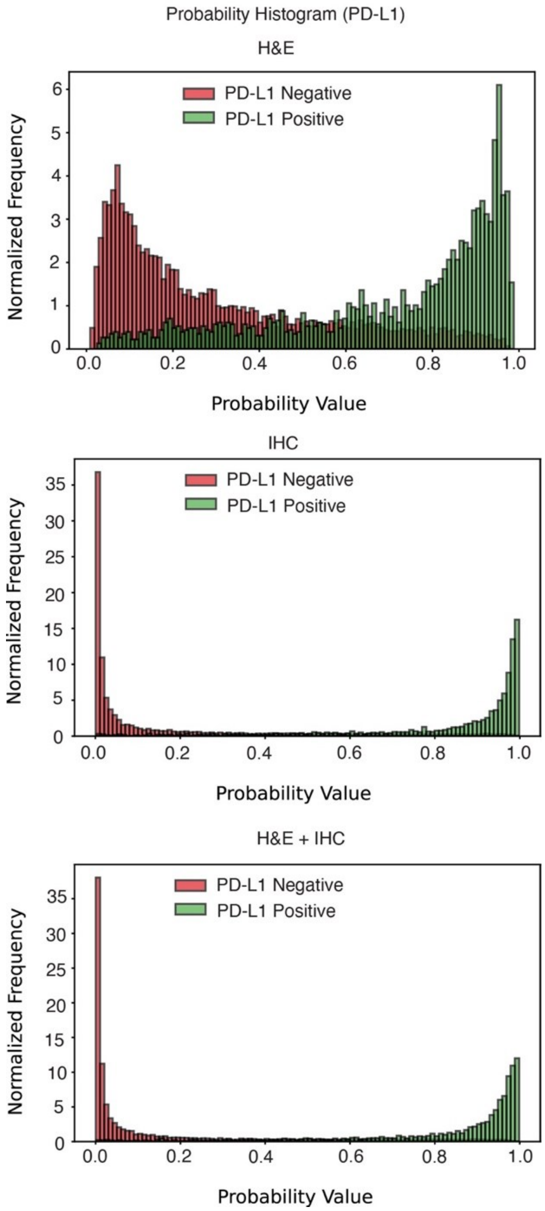

Supplement: Supplementary file 2 — Supplementary Information [file 43856_2025_1045_MOESM2_ESM.pdf]
