# Supplementary material for: Synergistic H&E and IHC image analysis by AI predicts cancer biomarkers and survival outcomes in colorectal and breast cancer
Source: Commun Med (Lond). 2025 Aug 1;5:328. doi: 10.1038/s43856-025-01045-9 (PMC12317095; doi:10.1038/s43856-025-01045-9)
Supplement: Supplementary file 3 — Description of Additional Supplementary Files [file 43856_2025_1045_MOESM3_ESM.pdf]

Description of Additional Supplementary files

File name: Supplementary Data 1

Description: Data used for the trade-off analysis and plotting based on MMRd prediction using the dual-modality model.

File name: Supplementary Data 2

Description: Data used for the trade-off analysis and plotting based on MSI prediction using the dual-modality model.

File name: Supplementary Data 3

Description: Data used for the trade-off analysis and plotting based on PD-L1 prediction using the dual-modality model.

File name: Supplementary Data 4

Description: Data used for the trade-off analysis based on MSI prediction using the H&E-only model.
