# Supplementary material for: Synergistic H&E and IHC image analysis by AI predicts cancer biomarkers and survival outcomes in colorectal and breast cancer
Source: Commun Med (Lond). 2025 Aug 1;5:328. doi: 10.1038/s43856-025-01045-9 (PMC12317095; doi:10.1038/s43856-025-01045-9)
Supplement: Supplementary file 8 — Reporting Summary [file 43856_2025_1045_MOESM8_ESM.pdf]

nature portfolio

Corresponding author(s): George W. Sledge, Jr
Last updated by author(s): 6.13.25



# Reporting Summary

Nature Portfolio wishes to improve the reproducibility of the work that we publish. This form provides structure for consistency and transparency in reporting. For further information on Nature Portfolio policies, see our Editorial Policies and the Editorial Policy Checklist.

## Statistics

For all statistical analyses, confirm that the following items are present in the figure legend, table legend, main text, or Methods section.

| n/a | Confirmed | |
|---|---|---|
| ☐ | ☒ | The exact sample size ($n$) for each experimental group/condition, given as a discrete number and unit of measurement |
| ☐ | ☒ | A statement on whether measurements were taken from distinct samples or whether the same sample was measured repeatedly |
| ☐ | ☒ | The statistical test(s) used AND whether they are one- or two-sided<br>*Only common tests should be described solely by name; describe more complex techniques in the Methods section.* |
| ☐ | ☒ | A description of all covariates tested |
| ☐ | ☒ | A description of any assumptions or corrections, such as tests of normality and adjustment for multiple comparisons |
| ☐ | ☒ | A full description of the statistical parameters including central tendency (e.g. means) or other basic estimates (e.g. regression coefficient) AND variation (e.g. standard deviation) or associated estimates of uncertainty (e.g. confidence intervals) |
| ☐ | ☒ | For null hypothesis testing, the test statistic (e.g. $F$, $t$, $r$) with confidence intervals, effect sizes, degrees of freedom and $P$ value noted<br>*Give P values as exact values whenever suitable.* |
| ☒ | ☐ | For Bayesian analysis, information on the choice of priors and Markov chain Monte Carlo settings |
| ☒ | ☐ | For hierarchical and complex designs, identification of the appropriate level for tests and full reporting of outcomes |
| ☒ | ☐ | Estimates of effect sizes (e.g. Cohen's $d$, Pearson's $r$), indicating how they were calculated |

*Our web collection on statistics for biologists contains articles on many of the points above.*

## Software and code

Policy information about availability of computer code

| | |
|---|---|
| Data collection | WSI processing and tile generation were done using QuPath (0.4.4). All deep learning experiments were done using Python (3.9) and PyTorch 1.13.1 . |
| Data analysis | Data analysis was performed using Python in an AWS SageMaker environment. We utilized the Docker container image pytorch-training:1.13.1-gpu-py39-cu117-ubuntu20.04-ec2. The code was developed based on the open-source project HistoBistro: https://github.com/peng-lab/HistoBistro. Detailed implementation is provided in the Methods section of the manuscript. Implementation has been described in the manuscript's method section. |

For manuscripts utilizing custom algorithms or software that are central to the research but not yet described in published literature, software must be made available to editors and reviewers. We strongly encourage code deposition in a community repository (e.g. GitHub). See the Nature Portfolio guidelines for submitting code & software for further information.

## Data

Policy information about availability of data

All manuscripts must include a data availability statement. This statement should provide the following information, where applicable:

- Accession codes, unique identifiers, or web links for publicly available datasets
- A description of any restrictions on data availability
- For clinical datasets or third party data, please ensure that the statement adheres to our policy

The data generated in this study are not publicly available due to data size and patient privacy but will be made available for replication and verification purposes from the corresponding author upon signing of a data usage agreement.

## Research involving human participants, their data, or biological material

Policy information about studies with human participants or human data. See also policy information about sex, gender (identity/presentation), and sexual orientation and race, ethnicity and racism.

| | |
|---|---|
| Reporting on sex and gender | Patient sex was reported for the cohorts in this study, but was not further analyzed. |
| Reporting on race, ethnicity, or other socially relevant groupings | Race and ethnicity information was not fully annotated or analyzed. |
| Population characteristics | Patients were analyzed based on having received pembrolizumab treatment, but further details on other clinical characteristics or sequence of other treatment regimens were not available. |
| Recruitment | Not applicable. This was a retrospective analysis. |
| Ethics oversight | This study was conducted in accordance with guidelines of the Declaration of Helsinki, Belmont Report, and US Common Rule. In keeping with 45 CFR 46.101(b)(4), this study was carried out utilizing retrospective, deidentified clinical data. Therefore, this study is considered exempt from institutional review board (IRB) approval and no patient consent was necessary. Exempt status was determined by wcg IRB. |

Note that full information on the approval of the study protocol must also be provided in the manuscript.

# Field-specific reporting

Please select the one below that is the best fit for your research. If you are not sure, read the appropriate sections before making your selection.

☒ Life sciences ☐ Behavioural & social sciences ☐ Ecological, evolutionary & environmental sciences

For a reference copy of the document with all sections, see nature.com/documents/nr-reporting-summary-flat.pdf

# Life sciences study design

All studies must disclose on these points even when the disclosure is negative.

| | |
|---|---|
| Sample size | This is a retrospective study. As such, sample sizes were determined based on availability in the Caris Life Sciences clinico-genomic database. |
| Data exclusions | Patients with insufficient outcomes data derived from insurance claims were excluded. |
| Replication | Testing our model on an independent dataset would be ideal; however, there currently appears to be no publicly available dataset that includes both H&E and IHC images for such use. |
| Randomization | Randomization was not applicable to our study. Patients were allocated to groups based on biomarker status. |
| Blinding | Blinding was not applicable to our study as the machine learning model was applied to assign biomarker status. |

# Reporting for specific materials, systems and methods

We require information from authors about some types of materials, experimental systems and methods used in many studies. Here, indicate whether each material, system or method listed is relevant to your study. If you are not sure if a list item applies to your research, read the appropriate section before selecting a response.

## Materials & experimental systems

| n/a | Involved in the study |
| --- | --- |
| ☐ | ☒ Antibodies |
| ☒ | ☐ Eukaryotic cell lines |
| ☒ | ☐ Palaeontology and archaeology |
| ☒ | ☐ Animals and other organisms |
| ☐ | ☒ Clinical data |
| ☒ | ☐ Dual use research of concern |
| ☒ | ☐ Plants |

## Methods

| n/a | Involved in the study |
| --- | --- |
| ☒ | ☐ ChIP-seq |
| ☒ | ☐ Flow cytometry |
| ☒ | ☐ MRI-based neuroimaging |

# Antibodies

Antibodies used

MMR protein expression was determined using specific antibody clones for MLH1 (M1 antibody), MSH2 (G2191129 antibody), MSH6 (44 antibody), and PMS2 (EPR3947 antibody; Ventana Medical Systems, Inc.).

The PD-L1 immunohistochemical staining was carried out on FFPE sections using the PD-L1 22C3 pharmDx kit, according to FDA standards.

Validation

The MMR panel by Ventana is FDA approved for detection of MMR status in CRC. Please see https://www.accessdata.fda.gov/cdrh_docs/reviews/DEN170030.pdf.

The PD-L1 22c3 antibody kit is a validated FDA-approved test. See https://www.agilent.com/en-us/product/pharmdx/pd-l1-ihc-22c3-pharmdx-overview.

# Clinical data

Policy information about clinical studies

All manuscripts should comply with the ICMJE guidelines for publication of clinical research and a completed CONSORT checklist must be included with all submissions.

Clinical trial registration

N/A

Study protocol

N/A

Data collection

Data was retrospectively analyzed from the Caris Life Sciences clinico-genomic database.

Outcomes

Time-on-treatment and overall survival were derived from insurance claims data.

# Plants

Seed stocks

*Report on the source of all seed stocks or other plant material used. If applicable, state the seed stock centre and catalogue number. If plant specimens were collected from the field, describe the collection location, date and sampling procedures.*

Novel plant genotypes

*Describe the methods by which all novel plant genotypes were produced. This includes those generated by transgenic approaches, gene editing, chemical/radiation-based mutagenesis and hybridization. For transgenic lines, describe the transformation method, the number of independent lines analyzed and the generation upon which experiments were performed. For gene-edited lines, describe the editor used, the endogenous sequence targeted for editing, the targeting guide RNA sequence (if applicable) and how the editor was applied.*

Authentication

*Describe any authentication procedures for each seed stock used or novel genotype generated. Describe any experiments used to assess the effect of a mutation and, where applicable, how potential secondary effects (e.g. second site T-DNA insertions, mosiacism, off-target gene editing) were examined.*
